# Genomic-Enabled Prediction in Maize Using Kernel Models with Genotype × Environment Interaction

**DOI:** 10.1534/g3.117.042341

**Published:** 2017-04-28

**Authors:** Massaine Bandeira e Sousa, Jaime Cuevas, Evellyn Giselly de Oliveira Couto, Paulino Pérez-Rodríguez, Diego Jarquín, Roberto Fritsche-Neto, Juan Burgueño, Jose Crossa

**Affiliations:** * Department of Genetics, Luiz de Queiroz College of Agriculture, University of São Paulo, Piracicaba, 13418-900 Brazil; † University of Quintana Roo, 77019 Chetumal, Mexico; ‡ Colegio de Postgraduados, CP 56230 Montecillos, 56230 D.F., México; § Agronomy and Horticulture Department, University of Nebraska, Lincoln, Nebraska 68583-0915; ** Biometrics and Statistics Unit, International Maize and Wheat Improvement Center (CIMMYT), 06600 D.F., México

**Keywords:** Genotype× Environment interaction (G×E), Genomic Best Linear Unbiased Predictor (GBLUP) linear kernel, Gaussian nonlinear kernel, Genomic Selection, GenPred, Shared Data Resources

## Abstract

Multi-environment trials are routinely conducted in plant breeding to select candidates for the next selection cycle. In this study, we compare the prediction accuracy of four developed genomic-enabled prediction models: (1) single-environment, main genotypic effect model (SM); (2) multi-environment, main genotypic effects model (MM); (3) multi-environment, single variance G×E deviation model (MDs); and (4) multi-environment, environment-specific variance G×E deviation model (MDe). Each of these four models were fitted using two kernel methods: a linear kernel Genomic Best Linear Unbiased Predictor, GBLUP (GB), and a nonlinear kernel Gaussian kernel (GK). The eight model-method combinations were applied to two extensive Brazilian maize data sets (HEL and USP data sets), having different numbers of maize hybrids evaluated in different environments for grain yield (GY), plant height (PH), and ear height (EH). Results show that the MDe and the MDs models fitted with the Gaussian kernel (MDe-GK, and MDs-GK) had the highest prediction accuracy. For GY in the HEL data set, the increase in prediction accuracy of SM-GK over SM-GB ranged from 9 to 32%. For the MM, MDs, and MDe models, the increase in prediction accuracy of GK over GB ranged from 9 to 49%. For GY in the USP data set, the increase in prediction accuracy of SM-GK over SM-GB ranged from 0 to 7%. For the MM, MDs, and MDe models, the increase in prediction accuracy of GK over GB ranged from 34 to 70%. For traits PH and EH, gains in prediction accuracy of models with GK compared to models with GB were smaller than those achieved in GY. Also, these gains in prediction accuracy decreased when a more difficult prediction problem was studied.

Genomic selection (GS) arose from the need to improve the prediction of complex traits based on dense marker information. It was first proposed in animal breeding (Meuwissen *et al.* 2001), and then applied to plant breeding (de los Campos *et al.* 2009; Crossa *et al.* 2010, 2011). Using GS, genomic breeding values are estimated as the sum of marker effects for unphenotyped individuals in the testing population. Several genomic prediction models were developed and applied to simulated and real plant breeding data (Bernardo and Yu 2007; Massman *et al.* 2013; de los Campos *et al.* 2009, 2013; Crossa *et al.* 2010, 2011; Pérez-Rodríguez *et al.* 2013; Beyene *et al.* 2015). In general, these studies showed good prediction accuracies for complex traits evaluated by means of random cross-validation (CV) partitions of the data.

Genomic-enabled prediction models were originally developed for a single trait in a single environment. However, multi-environment plant breeding trials are routinely conducted to estimate and take advantage of genotype × environment interaction (G×E). Therefore, to implement GS strategies in plant breeding, G×E needs to be estimated, modeled, and predicted. When genetic marker information is used for computing associations between individuals through the Genomic Relationships Matrix (GRM) G (Habier *et al.* 2007), this model is also referred to as Genomic Best Linear Unbiased Predictor (GBLUP) (VanRaden 2007, 2008). Burgueño *et al.* (2012) extended the GBLUP methodology to incorporate and model G×E effects. The Bayesian model of Jarquín *et al.* (2014) is another GBLUP extension that introduces main and interaction effects of markers and environmental covariables via covariance structures. Heslot *et al.* (2014) proposed using crop modeling for assessing genomic G×E. In general, studies have shown that modeling G×E can give substantial gains in prediction accuracy (Burgueño *et al.* 2012; Heslot *et al.* 2014; Jarquín *et al.* 2014; Crossa *et al.* 2016a,b; Cuevas *et al.* 2016a,b).

Recently, López-Cruz *et al.* (2015) proposed a GBLUP prediction model that explicitly models G×E and marker × environment interaction (M×E) where marker effects and genomic values are partitioned into components that are stable across environments (main effects) and others that are environment-specific (interactions). The model of López-Cruz *et al.* (2015) has advantages and disadvantages; the advantages are: (i) it can be easily implemented using existing software for GS, for example, BGLR (de los Campos and Pérez-Rodríguez 2016); and (ii) it can be implemented using both shrinkage methods (a ridge-regression type estimator) and variable (marker) selection methods. In this case, the M×E model can be employed not only for GS but also for genome-wide association analyses to identify genomic regions that contribute to stability and to interaction effects (Crossa *et al.* 2016b). Furthermore, in terms of reducing the models’ residual variance, the M×E model outperformed the more traditional single-environment and across-environment models for complex traits (López-Cruz *et al.* 2015; Crossa *et al.* 2016b). However, the M×E model is more efficient when applied to sets of environments that have positive correlations. This limitation arises because the genetic covariance between any pair of environments is represented by the variance of the main effect, which forces the covariance between pairs of environments to be positive (López-Cruz *et al.* 2015).

VanRaden (2008) first suggested models using a standard linear kernel, where the GBLUP is a linear model characterized by parameters related to additive quantitative genetics theory. However, complex traits are affected by nonlinearity effects between genotypes and phenotypes due to complex interactions among genes (*i.e.*, epistasis) and their interaction with the environment. Gianola *et al.* (2006) proposed nonparametric and semiparametric methods to model the relationship between the phenotype and markers that are available within the GS framework. The nonparametric methods are capable of accounting for small complex epistatic interactions without explicitly modeling them.

Therefore, the semi-parametric Reproducing Kernel Hilbert Space (RKHS) reduces the dimensions of the parametric space and also captures small complex interactions among markers (Gianola and van Kaam 2008; de los Campos *et al.* 2010; Morota and Gianola 2014; Gianola *et al.* 2006, 2014). The RKHS method uses a kernel function to convert the marker matrix into a set of distances between pairs of individuals (Heslot *et al.* 2012). Recently, Jiang and Reif (2015) formulated a model with explicit epistatic effects of markers and proved that this model is equivalent to RKHS with Gaussian kernel, thus demonstrating that this model captures epistatic effects among markers. Several studies confirmed the advantage of using RKHS regression to increase prediction accuracy by capturing nonadditive variation (de los Campos *et al.* 2010; Heslot *et al.* 2012; Morota *et al.* 2013; Pérez-Rodríguez *et al.* 2013; Morota and Gianola 2014).

Recently, Cuevas *et al.* (2016a) compared methods that applied the G×E interaction GS model of López-Cruz *et al.* (2015) using a linear kernel (GBLUP) and a nonlinear Gaussian kernel with the bandwidth estimated by an empirical Bayes method proposed by Pérez-Elizalde *et al.* (2015), and a Kernel Averaging method, or multi Gaussian kernels (de los Campos *et al.* 2010). Cuevas *et al.* (2016a) evaluated these methods using single-environment and multi-environment G×E interaction models to show the higher prediction ability of the Gaussian kernel models with the G×E model *vs.* the linear kernel with the G×E model. The most flexible Gaussian kernels captured major and complex effects of markers in addition to their interaction effects.

In genomic-enabled prediction, linear models consider genetic values as linear combinations of marker effects; therefore, GBLUP with G×E can also be fitted with the nonlinear Gaussian kernel. Similarly, the model of Jarquín *et al.* (2014) can also be used with the linear GBLUP kernel, and with the nonlinear Gaussian kernel (GK). However, so far, the model of Jarquín *et al.* (2014) has not been used with the GK, and its prediction accuracy has not been compared with the G×E with the GBLUP kernel of López-Cruz *et al.* (2015), or with the model of Jarquín *et al.* (2014) across environments, or when including G×E with the GBLUP kernel.

Therefore, the main objectives of this research were: (1) to study the prediction accuracy of the multi-environment, single variance G×E deviation model (MDs) of Jarquín *et al.* (2014) used with the GK method (MDs-GK) and the prediction accuracy of the multi-environment, environment-specific variance G×E deviation model (MDe) of López-Cruz *et al.* (2015) fitted with the GK method (MDe-GK), and to compare them with the prediction accuracy of their counterpart models using the linear kernel GBLUP (GB) method (MDs-GB and MDe-GB), and (2) to compare the accuracy of the four previous models with the accuracy of the single-environment, main genotypic effect model (SM), and the multi-environment, main genotypic effect (MM) of Jarquín *et al.* (2014) using GB and GK methods (SM-GB, SM-GK, MM-GB, and MM-GB).

Two data sets of Brazilian maize hybrids (HEL and USP) genotyped with dense molecular markers, and phenotyped in different environments with different numbers of hybrids per environment and different traits, were used to compare the prediction accuracy of those eight model-methods.

## Materials and Methods

### Phenotypic experimental data

This study considered two hybrid maize data sets. The first data set is from the Helix Seeds Company (HEL), while the second is from the University of São Paulo (USP). The HEL data set consists of 452 maize hybrids obtained by crossing 111 pure lines (inbreds); the hybrids were evaluated in 2015 at five Brazilian sites: Nova Mutum (NM), (13° 05′ S, 56° 05′ W, 460 m above sea level) and Sorriso (SO) (12° 32′ S, 55° 42′ W, 365 m above sea level) in the state of Mato Grosso; Pato de Minas (PM) (18° 34′ S, 46° 31′ W, 832 m above sea level), and Ipiaçú (IP) (18° 41′ S, 49° 56′ W, 452 m above sea level) in the state of Minas Gerais; and Sertanópolis (SE) (23° 03′ S, 51° 02′ W, 361 m above sea level) in the state of Parana. The experimental design was a randomized block with two replicates per genotype and environment. The phenotypic and genomic data on inbred lines are credited to Helix Seeds Ltda Company.

Data from USP consist of 740 maize hybrids obtained by crossing 49 inbred lines. The hybrids were evaluated at Piracicaba and Anhumas, São Paulo, Brazil, in 2016. The hybrids were evaluated using an augmented block design, with two commercial hybrids as checks to correct for micro-environmental variation. At each site, two levels of nitrogen (N) fertilization were used: Ideal N (IN) and Low N (LN) for a total of four artificial environments (P-IN, P-LN, A-IN, and A-IN). The experiment conducted under ideal N conditions received 100 kg ha^−1^ of N (30 kg ha^−1^ at sowing and 70 kg ha^−1^ in a coverage application) at the V8 plant stage, while the experiment with low N received 30 kg/ha of N at sowing. Each plot was 7 m in length, with 0.50 m spacing between rows and 0.33 m between plants.

**Figure 1:**
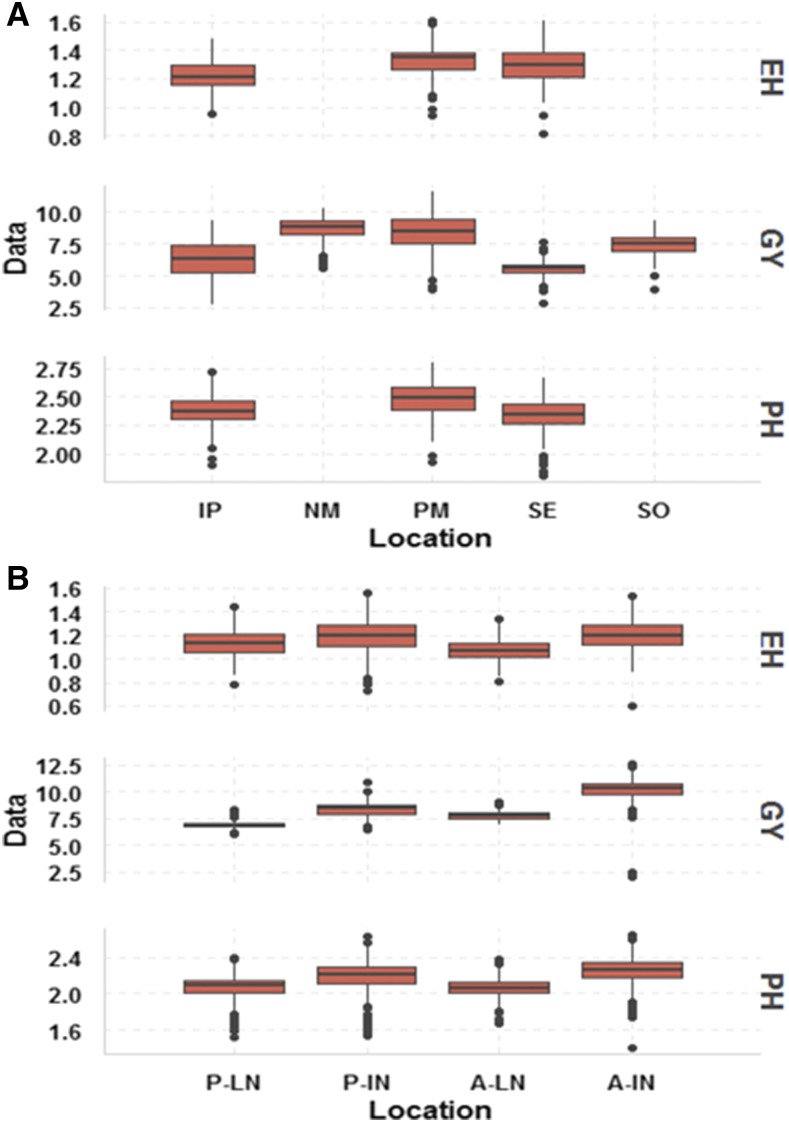
Box plot of grain yield (ton per hectare) (GY), plant height (centimeter) (PH), and ear height (centimeter) (EH) for (A) HEL data set and (B) USP data set. Environments for the HEL data set are IP, Ipiaçú; NM, Nova Mutum; PM, Pato de Minas; SE, Sertanópolis; and SO, Sorriso. Environments for the USP data set are: P-LN, Piracicaba low nitrogen; P-IN, Piracicaba ideal nitrogen; A-LN, Anhumas low nitrogen; and A-IN, Anhumas ideal nitrogen.

There was an imbalance in both data sets because not all hybrids were evaluated in all locations (incomplete field trials). The main traits of the two data sets were GY (grain yield in ton per hectare), PH (plant height in centimeters), and EH (ear height in centimeters). Plant height was measured from the ground to the flag leaf and EH from the ground to the base of the ear. The empirical distribution of the three evaluated traits (GY, PH, and EH) were symmetric in most of the environments (Figure 1). For the HEL data set, PH and EH were evaluated in three environments. Broad sense heritability (repeatability) was computed using the standard formula based on plot means:

$$\sigma_{h}^{2}/[\sigma_{h}^{2}+\sigma_{hs}^{2}/s+\sigma_{e}^{2}/sr],$$

where $\sigma_{h}^{2}$ is the variance of the hybrids, $\sigma_{hs}^{2}$ is the variance of the hybrids × location interaction and $\sigma_{e}^{2}\;$is the residual error variance and *s* and *r* are the number of environments and replicates in each environment, respectively.

### Genotypic data

The USP and HEL parent lines were genotyped with an Affymetrix Axiom Maize Genotyping Array of 616 K SNPs (Unterseer *et al.* 2014). Standard quality controls (QC) were applied to the data, removing markers with a Call Rate ≥ 0.95. The remaining missing data in lines were imputed with the Synbreed package (Wimmer *et al.* 2015) using the algorithms from the software Beagle 4.0 (Browning and Browning 2008). The hybrid genotypes were obtained by genomic information of the parent inbred lines. Markers with Minor Allele Frequency (MAF) of ≤0.05 were removed. After applying QC, 52,811 and 54,113 SNPs were available to make the predictions in the HEL and USP data sets, respectively.

## Statistical models

Statistical models for genomic predictions taking into account G×E were proposed by Jarquín *et al.* (2014) and López-Cruz *et al.* (2015). The Jarquín model incorporates genetic information from molecular markers or from pedigree (Pérez-Rodríguez *et al.* 2015), and/or from environmental covariates, whereas the López-Cruz model decomposes the marker effect across all environments and for each specific environment (interaction).

In this study, four statistical prediction models were fitted to both data sets to study their prediction accuracy using random CV schemes (Table 1). We also compared the prediction accuracy of two kernel regression methods in the four models. The models were: a single-environment, main genotypic effect model (SM), a multi-environment, main genotypic effect model (MM) (Jarquín *et al.* 2014), a multi-environment, single variance G×E deviation model (MDs) (Jarquín *et al.* 2014) and a multiple-environment environment-specific variance G×E deviation model (MDe) (López-Cruz *et al.* 2015). The two kernel regression methods were: the linear kernel GBLUP (GB) method used by Jarquín *et al.* (2014) and López-Cruz *et al.* (2015), and the Gaussian kernel (GK) method proposed by Cuevas *et al.* (2016a).

**Table 1:** Model, kernel method, and abbreviation of the model-method combination

Model*^a^*	Kernel Method	Abbreviation
Single-environment, main genotypic effect model (SM)	GBLUP (GB)	SM-GB
** $y=\mu 1+Z_{u}u+\varepsilon$**	Gaussian Kernel (GK)	SM-GK
Multi-environment, main genotypic effect (MM)	GBLUP (GB)	MM-GB
$y=\mu 1+Z_{E}\beta_{E}+Z_{u}u+\varepsilon$	Gaussian Kernel (GK)	MM-GK
Multi-environment, single variance G×E deviation model (MDs)	GBLUP (GB)	MDs-GB
** $y=\mu 1+Z_{E}\beta_{E}+Z_{u}u+\mathrm{ue}+\varepsilon$**	Gaussian Kernel (GK)	MDs-GK
Multi-environment, environment-specific variance G×E deviation model (MDe)	GBLUP (GB)	MDe-GB
** $y=\mu 1+Z_{E}\beta_{E}+Z_{u}u_{0}+u_{E}+\varepsilon$**	Gaussian Kernel (GK)	MDe-GK

a Model-methods are described in the section *Materials and Methods*.

### The single-environment, main genotypic effect model (SM)

The SM model fits the data from each environment separately and takes into account the main effect of genotypes. In matrix notation the model is written as:

$$y=\mu 1+Z_{u}u+\varepsilon$$ (1)

where $y={\left(y_{1},\ldots ,y_{n}\right)}^{\prime }$ is the response vector, and $y_{i}$ represents the observations in the *i*th line (i=1,…,n) in each environment; μ is the general mean; $Z_{u}$ is the incidence matrix that connects the random genetic effects with phenotypes; u is the random genetic effects for each environment, and ε the residual random effects for each environment. The SM model (1) assumes that the distribution of the u vector is multivariate normal with mean zero and a covariance matrix $\sigma_{u_{j}}^{2}K,$ that is, $u∼N\left(0,\sigma_{u_{j}}^{2}K\right),$ where $\sigma_{u_{j}}^{2}$ is the genetic variance component of u in the *j*th environment and K is a symmetric, positive semi-definite matrix, denoting the variance–covariance of the genetic values constructed from the genomic molecular markers. It is also assumed that the errors ε in each environment are independent with homogeneous variance, $\sigma_{\varepsilon }^{2},$ and distributed as $\varepsilon ∼N\left(0,\;\sigma_{\varepsilon_{j}}^{2}I\right)$ (where ***I*** is the identity matrix, and $\sigma_{\varepsilon_{j}}^{2}$ is the residual variance in the *j*^th^ environment). Therefore, u is an approximation of the true unknown genetic values, and ε captures the residual genetic effects that were not explained by u plus other nongenetic effects that approximate the errors. Note that matrix ***K*** may have different dimensions depending on the number of lines evaluated in each environment.

### The single-environment, main genotypic effect model with GBLUP (SM-GB) and Gaussian kernel (SM-GK)

For SM model (1), matrix K can be constructed using the linear kernel ***K*** = (***XX***′/*p*) (de los Campos *et al.* 2013) proposed by VanRaden (2007, 2008) for estimating the GBLUP, where X is the standardized matrix of molecular markers for the individuals, of order n×p, where p is the number of markers (Table 1).

When markers have a more complex function, the GK has proved to be more effective for capturing complex cryptic interactions between markers, thereby improving the prediction accuracy of the model (Cuevas *et al.* 2016a). The entries of the Gaussian kernel are computed as $K\left(x_{i}x_{i^{\prime }}\right)=\exp\left(-hd_{ii^{\prime }}^{2}\right),$ where $d_{ii^{\prime }}$ is the Euclidean distance between the individuals ith and ${i^{\prime }}^{\text{th}}\;\left(i=1,\ldots ,\;n_{j}\right)$ given by the markers; h>0 is the bandwidth parameter that controls the rate of decay of K values (Pérez-Rodríguez *et al.*, 2012; Cuevas *et al.* 2016a). In this study, GK takes the form $K\left(x_{i}x_{i^{\prime }}\right)=\exp\left(-hd_{ii^{\prime }}^{2}/\text{median}\left(d_{ii^{\prime }}^{2}\right)\right),$ where h=1 and the median of the distances is used as a scaling factor (Crossa *et al.* 2010). de los Campos *et al.* (2010) described the theory of the Gaussian kernel in the context of the kernel averaging method for the RKHS.

### The multi-environment, main genotypic effect model (MM)

One multi-environment model considers the main fixed effects of environments, as well as the random genetic effects across environments

$$y=\mu 1+Z_{E}\beta_{E}+Z_{u}u+\varepsilon$$ (2)

where $y={\left(y_{1},\ldots ,y_{j},\ldots ,y_{s}\right)}^{\prime }$is the response vector and $y_{j}$ is the vector of observations of the lines $\left(i=1,\ldots ,\;n_{j}\right)$ in the *j*th environment (j=1,…,s). The fixed effects of the environment for the data used in this study are modeled with the incidence matrix of the environments $Z_{E},$ where the parameters to be estimated are the intercept for each environment ($\beta_{E}$) (other fixed effects can be incorporated into the model), and incidence matrix $Z_{u}$ connects genotypes with phenotypes for each environment. It is assumed that the random vector of genetic effects u across environments follows a multivariate normal with mean zero and a covariance matrix $\sigma_{u_{0}}^{2}K,$ that is, $u∼N\left(0,\sigma_{u_{0}}^{2}K\right),$ where $\sigma_{u_{0}}^{2}$ is the variance of the main genetic effects across environments, and $\varepsilon ∼N(0,\;\sigma_{\varepsilon }^{2}I).$ In this case, the resulting model is equivalent to the across-environment GBLUP model of López-Cruz *et al.* (2015) and Jarquín *et al.* (2014). When in the MM model of (2) u is used with ***K*** = (***XX***′/*p*) (GBLUP), the model is the GBLUP across environments (MM-GB), whereas if u is used with the GK, the model is the GK across environments (MM-GK) (Table 1).

### Multi-environment, single variance G×E deviation model (MDs)

This model extends (2) by adding the random interaction effect of the environments with the genetic information of the lines (ue)

$$y=\mu 1+Z_{E}\beta_{E}+Z_{u}u+\mathrm{ue}+\varepsilon$$ (3)

It is assumed that the vector of random effects of the interaction ue has a multivariate normal distribution, $\mathrm{ue}∼N\left(0,\left[Z_{u}KZ_{u}^{\prime }\right]\circ \left[Z_{E}Z_{E}^{\prime }\right]\sigma_{ue}^{2}\right),$ where (∘) is the Haddamar product operator and denotes the element-to-element product between two matrices in the same order (Jarquín *et al.* 2014), $\sigma_{ue}^{2}$ is the variance component of the interaction, the K matrix is defined as before, and u is the vector of the main genetic effects that follows a multivariate normal with mean zero and a covariance matrix $\sigma_{u_{0}}^{2}K,$ that is, $u∼N\left(0,\sigma_{u_{0}}^{2}K\right),$ where $\sigma_{u_{0}}^{2}$ is the variance of the main genetic effects and $\varepsilon ∼N(0,\sigma_{\varepsilon }^{2}I).$

Therefore, under these conditions

$$\left[Z_{u}KZ_{u}^{\prime }\right]\circ \left[Z_{E}Z_{E}^{\prime }\right]=\left[\begin{array}{ccccc} K_{1} & \cdots & 0 & \cdots & 0\\ \vdots & \ddots & \vdots & \ddots & \vdots \\ 0 & \cdots & K_{j} & \cdots & 0\\ \vdots & \ddots & \vdots & \ddots & \vdots \\ 0 & \cdots & 0 & \cdots & K_{m} \end{array}\right]$$

where $K_{j}$ represents the kernel constructed from the molecular markers of the lines in the *j*th environment. Just as in model (2), matrix K is used in the variance–covariance for **u** of model (3), and is also a component of the variance-covariance of ue. Then, kernel matrix K can be constructed using the linear kernel (MDs-GB) or the GK (MDs-GK) (Table 1).

### Multi-environment, environment-specific variance G×E deviation model (MDe)

The model of López-Cruz *et al.* (2015) separates the genetic effects of markers into the main marker effects across all environments and the specific marker effects in each environment. Thus, this model in matrix notation is

$$y=\mu 1+Z_{E}\beta_{E}+Z_{u}u_{0}+u_{E}+\varepsilon$$ (4)

For the MDe model (4) of the vector, $u_{0}$ represents the main effect of markers (across all environments) with a variance–covariance structure similar to those used in models (2) and (3), that is, $u_{0}∼N\left(0,\sigma_{u_{0}}^{2}K\right).$ However, as pointed out by López-Cruz *et al.* (2015) and Cuevas *et al.* (2016a), $\sigma_{u0}^{2}$ is common to all *s* environments, and the borrowing of information among environments is generated through kernel matrix K. On the other hand, $u_{E}$ represents the specific effect of the markers in environments (or the effects of the interaction) with a variance–covariance structure different from model (3), that is, $u_{E}∼N\left(0,K_{E}\right),$ where $K_{E}$ is:

$$\begin{array}{c} K_{E}=\left[\begin{array}{ccccc} \sigma_{u_{E_{1}}}^{2}K_{1} & \cdots & 0 & \cdots & 0\\ \vdots & \ddots & \vdots & \ddots & \vdots \\ 0 & \cdots & \sigma_{u_{E_{j}}}^{2}K_{j} & \cdots & 0\\ \vdots & \ddots & \vdots & \ddots & \vdots \\ 0 & \cdots & 0 & \cdots & \sigma_{u_{E_{m}}}^{2}K_{m} \end{array}\right]=\\ \left[\begin{array}{ccccc} \sigma_{u_{E_{1}}}^{2}K_{1} & \cdots & 0 & \cdots & 0\\ \vdots & \ddots & \vdots & \ddots & \vdots \\ 0 & \cdots & 0 & \cdots & 0\\ \vdots & \ddots & \vdots & \ddots & \vdots \\ 0 & \cdots & 0 & \cdots & 0 \end{array}\right]+\cdots +\left[\begin{array}{ccccc} 0 & \cdots & 0 & \cdots & 0\\ \vdots & \ddots & \vdots & \ddots & \vdots \\ 0 & \cdots & \sigma_{u_{E_{j}}}^{2}K_{j} & \cdots & 0\\ \vdots & \ddots & \vdots & \ddots & \vdots \\ 0 & \cdots & 0 & \cdots & 0 \end{array}\right]+\cdots +\left[\begin{array}{ccccc} 0 & \cdots & 0 & \cdots & 0\\ \vdots & \ddots & \vdots & \ddots & \vdots \\ 0 & \cdots & 0 & \cdots & 0\\ \vdots & \ddots & \vdots & \ddots & \vdots \\ 0 & \cdots & 0 & \cdots & \sigma_{u_{E_{s}}}^{2}K_{s} \end{array}\right] \end{array}$$

Note that matrix $K_{E}$ can be expressed as the sum of **s** matrices, and the effects given by $u_{Ej}$ are specific for the jth environment; thus, it has a normal distribution with mean and variance equal to **0**, except for the jth environment, which has a variance–covariance matrix $\sigma_{uE_{j}}^{2}K_{j}.$ The importance of these two terms ($u\;\text{and}\;u_{E}$) of MDe model (4) is given by the corresponding variance components that are estimated from the data. Kernel matrix K is used in the components of u, while kernel matrix $K_{E}$ is used in the component of $u_{E};\;$both, K and $K_{E}$ can be used with linear kernel (MDe-GB) or with the Gaussian kernel (MDe-GK) (Table 1).

### Estimating variance components using full data analyses

The four models—SM, MM, MDs, and MDe fitted with GBLUP and GK methods—were used on the entire HEL and USP data sets for all the traits. To fit the models, the phenotypic data were centered and standardized (*i.e.*, each phenotypic data point was centered by subtracting the overall mean of all environments and standardized by dividing by the sample standard deviation across all environments). These analyses were performed to derive estimates of variance components. The posterior variance components resulting from the residual effects, genetic main effect, and genetic environment-specific effects of the four models described above for three traits and for environments in HEL and USP data sets were computed and reported. Since the data were standardized for GBLUP method, the summation of the variance components approximates 1.

### Assessing prediction accuracy by random CV

The prediction accuracy of the single-environment model-methods was assessed using 50 random partitions, organized in five folds with 10 random partitions each, with 80% of the hybrids comprising the training (TRN) set, and the remaining 20% of the individuals comprising the testing (TST) set. This was performed separately in each environment, as the single-environment models were fitted separately for each environment. For this validation procedure, all the parameters of the models (including variance components resulting from residual effects, and genetic effects) were re-estimated from TRN data in each of the TRN-TST partitions

For the multi-environment models, the prediction accuracy of the model-methods for predicting the pattern of missing values in each environment was generated using two different CV designs (Burgueño *et al.* 2012). The random CV1 design mimics the prediction problem faced by breeders when newly developed lines have not been evaluated in any environment; in this case, 20% of the lines were not observed (unphenotyped) in all the environments and had to be predicted. The random CV2 design mimics a prediction problem when lines are tested in incomplete field trials (sparse testing), where some lines are evaluated in some environments but not included in other environments. For these approaches, we assigned 80% of the observations to TRN and 20% to TST. None of the lines to be predicted in the TST are in the TRN set. For this validation procedure, all the parameters of the models (including variance components resulting from residual effects, genetic main effects, genetic × environment interactions effects, and environment-specific effects) were re-estimated from TRN data in each of the 50 random TRN-TST partitions. Note that CV2 design can only be applied to multi-environment model-methods (MM, MDs, and MDe), but not to single-environment model-methods (SM). Also, for SM the random CV is a CV1 but applied to only one individual environment (site).

For each TRN-TST partition, models were fitted to the TRN data set, and prediction accuracy was assessed by computing Pearson’s product–moment correlation between predictions and phenotypes in the TST data set within environments. The same TRN-TST partitions were used to assess the prediction accuracy of each of the models. Thus, 50 correlations were computed for each model and trait; the mean and SDs of these 50 correlations are reported.

Therefore, adjusting models for full data, making inference on the parameters, and assessing predictions in TRN-TST partitions were based on 30,000 samples collected from the posterior and predictive distributions after discarding 5000 samples as burn-in.

### Software

The aforementioned models can be implemented using the R (R Core Team 2016) package Bayesian generalized linear regression (BGLR) (de los Campos and Pérez-Rodríguez 2016; Pérez-Rodríguez and de los Campos 2014). Appendix A gives the R codes used in the models included in this study when the numbers of individuals in different environments vary. It should be pointed out that, in previous studies using the Jarquín *et al.* (2014) and López-Cruz *et al.* (2015) models, the number of entries in each environment was the same; thus, the model fitting and the random CV partition scheme were simpler than in this case, where there are different numbers of entries in different environments.

### Data availability

The phenotypic and genotypic data for the maize hybrids included in this study can be found at http://hdl.handle.net/11529/10887. Each HEL and USP data set contains the data corresponding to each GB and GK kernel, as well as phenotypic data for each trait (PH, EH, and GY) in each environment.

## Results

### Descriptive statistics

Box-plots of GY, PH, and EH in each environment for the two data sets are depicted in Figure 1. Most of the distributions of the traits in environments had a symmetric distribution. For the HEL data set, environments NM and PM produced better GY, while environment SE had the lowest GY (Figure 1A). Traits PH and EH showed similar trends, but were measured in only three environments: IP, PM, and SE. Data from USP had environments with soil nutritional stress, and the results showed that environments with ideal nitrogen conditions (P-IN and A-IN) had better potential for all traits (Figure 1A).

All pair-wise environments for all traits in the HEL and USP data sets had high and positive correlations (Table 2 and Table 3). As expected, for PH and EH, the correlations were higher than for the complex trait GY. In all environments, GY, PH, and EH showed high heritability in the HEL data set: GY ranged from 0.60 to 0.86, PH varied from 0.72 to 0.91, and EH ranged from 0.69 to 0.89. For the USP data set, heritability values were high for PH and EH in all environments (from 0.60 to 0.85 and 0.66 to 0.91, respectively). For the USP data set, trait GY had an intermediate value of 0.42 for sites Anhumas and Piracicaba with ideal nitrogen (IN) and lower values for Piracicaba and Anhumas under low nitrogen conditions (LN) (0.22 and 0.19, respectively).

**Table 2:** Heritability and phenotypic correlations among five environments for grain yield, and three environments for plant height and ear height for the HEL data set

	Ipiaçú	Nova Mutum	Pato de Minas	Sertanópolis	Sorriso
Site	Grain yield				
Nova Mutum	0.46	—	—	—	—
Pato de Minas	0.51	0.44	—	—	—
Sertanópolis	0.29	0.36	0.30	—	—
Sorriso	0.43	0.48	0.39	0.38	—
	Lower diagonal, plant height; upper diagonal, ear height				

Ipiaçú	—	—	0.74	0.73	—
Pato de Minas	0.73	—	—	0.73	—
Sertanópolis	0.73	—	0.74	—	—
Trait	Heritability				
Grain yield	0.81	0.60	0.86	0.69	0.78
Plant height	0.72	—	0.86	0.91	—
Ear height	0.69	—	0.81	0.89	—

**Table 3:** Heritability and phenotypic correlations among four environments for grain yield, plant height, and ear height for the USP data set

	Piracicaba-LN*^a^*	Piracicaba-IN	Anhumas-LN	Anhumas-IN
Environment	Grain yield			
Piracicaba-IN	0.54	—	—	—
Anhumas-LN	0.31	0.35	—	—
Anhumas-IN	0.43	0.47	0.47	—
	Lower diagonal, plant height; upper diagonal, ear height			
Piracicaba-LN	—	0.80	0.71	0.78
Piracicaba-IN	0.75	—	0.69	0.78
Anhumas-LN	0.68	0.67	—	0.71
Anhumas-IN	0.76	0.78	0.70	—
Trait	Heritability			
Grain yield	0.22	0.42	0.19	0.42
Plant height	0.72	0.85	0.60	0.84
Ear height	0.73	0.87	0.66	0.91

a LN, low nitrogen; IN, ideal nitrogen.

### Estimating variance components

The distribution of the residuals after fitting the eight model-method combinations to the two data sets was approximately normal (data not shown). Phenotypic (and marker) data were standardized; thus summation of variance components approximated 1 for GB models. Deviations may be due to the high degree of imbalance in the number of hybrids in different environments, especially for data set HEL.

### HEL data set

#### Single-environment:

For the SM model for the three traits in each environment, the estimated residual variance components for the GK method were smaller than those for the GB method (Table 4). In contrast, the variance component of genetic effects for each environment increased for the SM-GK model-method as compared to the genetic effects of model-method SM-GB.

**Table 4:** HEL data set

		Grain Yield		Plant Height		Ear Height	
Component	Environment	SM-GB	SM-GK	SM-GB	SM-GK	SM-GB	SM-GK
Single-environment, main genotypic effect (SM)							
Residual ($\sigma_{\varepsilon_{j}}^{2}$)	Ipiaçú	0.476 (0.05)	0.259 (0.05)	0.397 (0.05)	0.243 (0.05)	0.419 (0.05)	0.246 (0.05)
	Nova Mutum	0.715 (0.06)	0.431 (0.07)	—	—	—	—
	Pato de Minas	0.440 (0.03)	0.221 (0.03)	0.332 (0.03)	0.151 (0.02)	0.330 (0.03)	0.163 (0.02)
	Sertanópolis	0.761 (0.07)	0.467 (0.07)	0.341 (0.05)	0.198 (0.03)	0.387 (0.03)	0.225 (0.03)
	Sorriso	0.742 (0.07)	0.467 (0.07)	—	—	—	—
Genetic effect ($\sigma_{u_{j}}^{2}$)	Ipiaçú	0.442 (0.10)	0.965 (0.13)	0.794 (0.17)	1.231 (0.23)	0.726 (0.18)	1.221 (0.20)
	Nova Mutum	0.391 (0.12)	1.177 (0.27)	—	—	—	—
	Pato de Minas	0.462 (0.09)	1.106 (0.15)	0.884 (0.16)	1.331 (0.15)	0.903 (0.16)	1.278 (0.15)
	Sertanópolis	0.290 (0.09)	1.156 (0.27)	0.855 (0.14)	1.224 (0.17)	0.736 (0.16)	1.199 (0.17)
	Sorriso	0.299 (0.09)	1.132 (0.25)	—	—	—	—
Multi-environment, main genotypic effects (MM)							
		MM-GB	MM-GK	MM-GB	MM-GK	MM-GB	MM-GK
Residual ($\sigma_{\varepsilon }^{2}$)		0.336 (0.01)	0.273 (0.01)	0.324 (0.02)	0.259 (0.01)	0.299 (0.013)	0.222 (0.01)
Genetic main effect ($\sigma_{u_{0}}^{2}$)		0.129 (0.03)	0.316 (0.041)	0.758 (0.12)	0.769 (0.08)	0.802 (0.13)	0.846 (0.08)
Multi-environment, single variance G×E deviation (MDs)							
		MDs-GB	MDs-GK	MDs-GB	MDs-GK	MDs-GB	MDs-GK
Residual ($\sigma_{\varepsilon }^{2}$)		0.230 (0.00)	0.107 (0.01)	0.274 (0.013)	0.164 (0.01)	0.294 (0.014)	0.188 (0.01)
Genetic main effect ($\sigma_{u_{0}}^{2}$)		0.122 (0.030)	0.375 (0.04)	0.798 (0.13)	0.844 (0.08)	0.763 (0.13)	0.764 (0.08)
Genetic Interaction effect ($\sigma_{ue}^{2}$)		0.111 (0.014)	0.244 (0.24)	0.04 (0.01)	0.124 (0.03)	0.051 (0.01)	0.155 (0.01)
Multi-environment, environment-specific variance G×E deviation (MDe)							
		MDe-GB	MDe-GK	MDe-GB	MDe-GK	MDe-GB	MDe-GK
Residual ($\sigma_{\varepsilon }^{2}$)		0.227 (0.01)	0.093 (0.01)	0.293 (0.01)	0.161 (0.01)	0.274 (0.01)	0.162 (0.01)
Genetic main effect ($\sigma_{u_{0}}^{2}$)		0.07 (0.02)	0.264 (0.03)	0.747 (0.12	0.834 (0.08)	0.798 (0.13)	0.837 (0.08)
Genetic environment specific effect ($\sigma_{uE_{j}}^{2}$)	Ipiaçú	0.278 (0.07)	0.658 (0.11)	0.046 (0.02)	0.096 (0.04)	0.040 (0.02)	0.09 (0.03)
	Nova Mutum	0.024 (0.01)	0.103 (0.03)	—	—	—	—
	Pato de Minas	0.32 (0.07)	0.792 (0.105)	0.036 (0.01)	0.091 (0.03)	0.034 (0.02)	0.090 (0.03)
	Sertanópolis	0.018 (0.01)	0.052 (0.02)	0.08 (0.03)	0.22 (0.07)	0.054 (0.03)	0.210 (0.07)
	Sorriso	0.023 (0.01)	0.077 (0.023)	—	—	—	—

Estimates of different variance components for single-environment, main genotypic effect GBLUP kernel (SM-GB), single-environment, main genotypic effect GK (SM-GK), multi-environment, main genotypic effect GBLUP kernel (MM-GB), multi-environment, main genotypic effect GK (MM-GK), multi-environment, single variance G×E deviation model GBLUP kernel (MDs-GB), multi-environment, single variance G×E deviation model GK (MDs-GK), multi-environment, environment-specific variance G×E deviation model GBLUP kernel (MDe-GB), multi-environment, environment-specific variance G×E deviation model GK (MDe-GK) for three traits: grain yield, plant height and ear height.

#### Multi-environment:

For the MM model, the residual variance components for MM-GK were smaller than the residuals for MM-GB for all traits, whereas, for the genetic effects, the opposite occurred: the variance component of MM-GK was higher than that of MM-GB. For the MDs models, the residual variance components for MDs-GK were always smaller than the residual variances for MDs-GB, whereas the opposite occurred for the genetic main effect and genetic interaction effect (Table 4).

The results of the MM and MDs models indicated that the inclusion of the interaction term (G×E) induced a larger reduction in the estimated residual variance for GY; for traits PH and EH, these reductions in residual variance were smaller than those found for GY from the MM and MDs models. For the MDe model, the residual variance components of MDe-GK were smaller than those of the MDe-GB for all traits, whereas the variance components for the genetic main effect and genetic environment specific effect were higher for the GK models than for the GB model. The genetic main effect values are related to the correlations between environments, which are positive and range from medium to high. For the variance component associated with the genetic interaction effect, the values are lower because they decreased G×E in environments with a positive correlation. In general, the fit of MDs is slightly worse than the fit of MDe models because the genetic interaction effect component has a small effect on all traits, but mainly on EH and PH (Table 4).

### USP data set

#### Single-environment:

For all traits, the variance components of genetic effects were higher when using the GK than the GB method in all environments and traits for the single-environment, main genotypic effect model (SM) (Table 5). The residual variance for SM-GK was always smaller than the residual for SM-GB for all traits.

**Table 5:** USP data set

Component	Environment*^a^*	Grain Yield		Plant Height		Ear Height	
		SM-GB	SM-GK	SM-GB	SM-GK	SM-GB	SM-GK
Single-environment, main genotypic effect (SM)							
Residual ($\sigma_{\varepsilon_{j}}^{2}$)	P-LN	0.878 (0.04)	0.797 (0.05)	0.749 (0.04)	0.656 (0.04)	0.503 (0.03)	0.377 (0.03)
	P-IN	0.851 (0.05)	0.786 (0.05)	0.685 (0.04)	0.594 (0.04)	0.492 (0.03)	0.390 (0.03)
	A-LN	0.873 (0.05)	0.817 (0.05)	0.812 (0.04)	0.726 (0.05)	0.627 (0.03)	0.513 (0.04)
	A-IN	0.780 (0.04)	0.699 (0.05)	0.654 (0.03)	0.543 (0.04)	0.494 (0.03)	0.375 (0.03)
Genetic effect ($\sigma_{u_{j}}^{2}$)	P-LN	0.132 (0.04)	0.438 (0.12)	0.257 (0.07)	0.619 (0.13)	0.556 (0.13)	0.955 (0.15)
	P-IN	0.149 (0.05)	0.420 (0.10)	0.341 (0.08)	0.683 (0.14)	0.537 (0.12)	0.829 (0.15)
	A-LN	0.124 (0.04)	0.366 (0.09)	0.188 (0.05)	0.523 (0.14)	0.397 (0.10)	0.808 (0.07)
	A-IN	0.232 (0.06)	0.550 (0.12)	0.383 (0.09)	0.747 (0.14)	0.549 (0.12)	0.922 (0.08)
Multi-environment, main genotypic effects (MM)							
		MM-GB	MM-GK	MM-GB	MM-GK	MM-GB	MM-GK
Residual ($\sigma_{\varepsilon }^{2}$)		0.864 (0.02)	0.656 (0.02)	0.705 (0.02)	0.319 (0.01)	0.519 (0.01)	0.279 (0.01)
Genetic main effect ($\sigma_{u_{0}}^{2}$)		0.167 (0..04)	1.39 (0.16)	0.341 (0.07)	3.131 (0.22)	0.56 (0.12)	1.861 (0.14)
Multi-environment, single variance G×E deviation (MDs)							
		MDs-GB	MDs-GK	MDs-GB	MDs-GK	MDs-GB	MDs-GK
Residual ($\sigma_{\varepsilon }^{2}$)		0.836 (0.02)	0.536 (0.02)	0.699 (0.02)	0.304 (0.01)	0.509 (0.01)	0.264 (0.01)
Genetic main effect ($\sigma_{u_{0}}^{2}$)		0.170 (0.04)	1.965 (0.18)	0.343 (0.08)	3.09 (0.21)	0.547 (0.12)	1.819 (0.13)
Genetic interaction effect ($\sigma_{ue}^{2}$)		0.024 (0.01)	0.094 (0.02)	0.008 (0.00)	0.057 (0.01)	0.019 (0.01)	0.073 (0.01)
Multi-environment, environment-specific variance G×E deviation (MDe)							
		MDe-GB	MDe-GK	MDe-GB	MDe-GK	MDe-GB	MDe-GK
Residual ($\sigma_{\varepsilon }^{2}$)		0.840 (0.02)	0.536 (0.02)	0.705 (0.02)	0.305 (0.01)	0.517 (0.01)	0.261 (0.01)
Genetic main effect ($\sigma_{u_{0}}^{2}$)		0.170 (0.04)	1.966 (0.19)	0.340 (0.08)	3.168 (0.22)	0.558 (0.12)	1.894 (0.13)
Genetic environment specific effect ($\sigma_{uE_{j}}^{2}$)	P-LN	0.013 (0.01)	0.110 (0.04)	0.006 (0.00)	0.042 (0.01)	0.006 (0.00)	0.043 (0.01)
	P-IN	0.016 (0.01)	0.081 (0.03)	0.005 (0.000)	0.045 (0.01)	0.006 (0.00)	0.039 (0.01)
	A-LN	0.014 (0.01)	0.101 (0.04)	0.004 (0.00)	0.058 (0.02)	0.008 (0.00)	0.09 (0.03)
	A-IN	0.020 (0.02)	0.087 (0.03)	0.004 (0.00)	0.037 (0.01)	0.006 (0.00)	0.039 (0.01)

Estimates of different variance components for single-environment, main genotypic effect GBLUP kernel (SM-GB), single-environment, main genotypic effect GK (SM-GK), multi-environment, main genotypic effect model GBLUP kernel (MM-GB), multi-environment, main genotypic effect GK (MM-GK), multi-environment, single variance G×E deviation model GBLUP kernel (MDs-GB), multi-environment, single variance G×E deviation model GK (MDs-GK), multi-environment, environment-specific variance G×E deviation model GBLUP kernel (MDe-GB), multi-environment, environment-specific variance G×E deviation model GK (MDe-GK) for three traits: grain yield, plant height and ear height

a Environments: A-IN, Anhumas ideal N; A-LN, Anhumas low N; P-IN, Piracicaba ideal N; P-LN Piracicaba low N.

#### Multi-environment:

The residual variance components of the MM and MDs models for all traits were very similar, as were most of the variance components for the genetic main effects. For all the environments and traits, the residuals from the GK were smaller than the residuals from the GB for the MM, MDs, and MDe models. The variance components of the genetic main effects of MDs-GB and MDe-GB were consistently smaller than the genetic main effect of model-methods MDs-GK and MDe-GK. Also, the variance components of the genetic environment specific effects of the GK models were higher than those of the GB models for all traits (Table 5).

### Prediction accuracy of the models with GBLUP and GK methods

**Figure 2:**
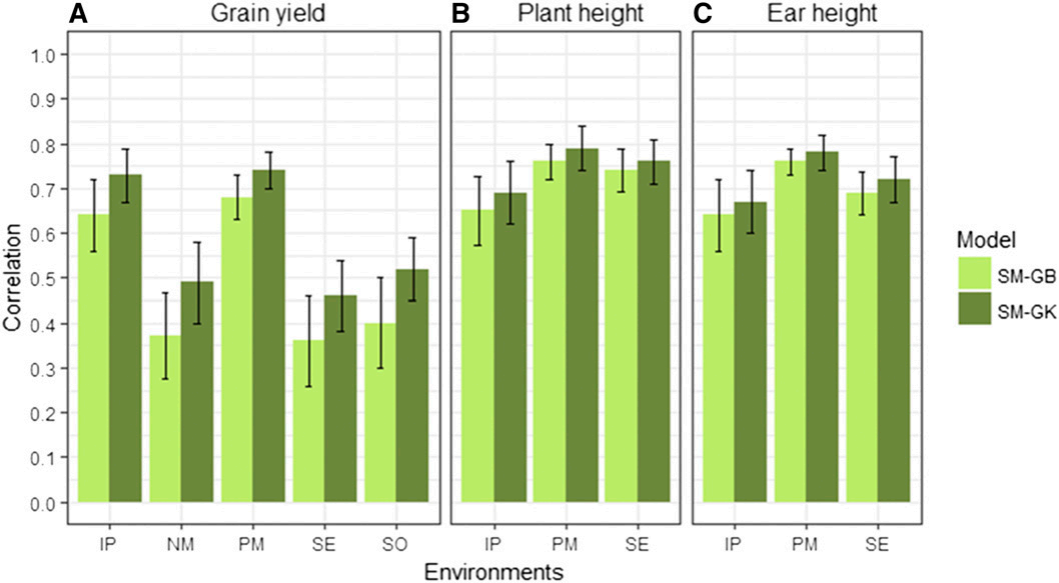
HEL data set. Correlation between phenotypes and prediction values (average of 50 random CV partitions) for single-environment, main genotypic effect model with GBLUP kernel method (SM-GB), and single-environment, main genotypic effect model with GK method (SM-GK) for: (A) five environments (horizontal axis) for grain yield, (B) three environments for plant height, and (C) three environments for ear height. Environments are: Ipiaçú (IP), Nova Mutum (NM), Pato de Minas (PM), Sertanópolis (SE) and Sorriso (SO). Error bars show SD.

**Figure 3:**
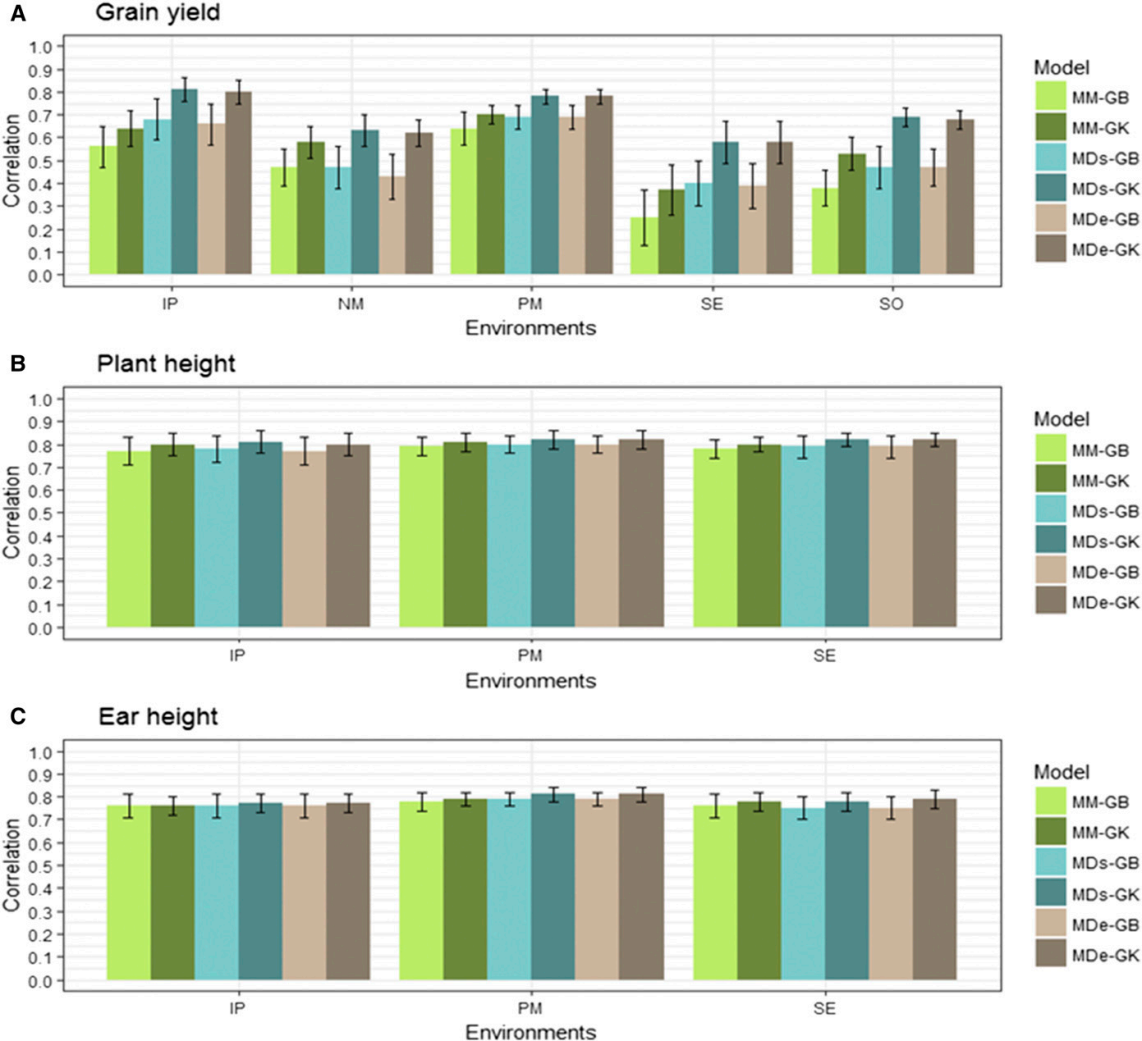
HEL data set. Mean correlation between observed and predictive values (average of 50 random CV partitions, CV2) for multi-environment, main genotypic effect model GBLUP kernel (MM-GB), multi-environment, main genotypic effect GK (MM-GK), multi-environment, single variance G×E deviation model GBLUP kernel (MDs-GB), multi-environment, single variance G×E deviation model GK (MDs-GK), multi-environment, environment-specific variance G×E deviation model GBLUP kernel (MDe-GB), multi-environment, environment-specific variance G×E deviation model GK (MDe-GK) for (A) five environments (horizontal axis) for grain yield, (B) three environments for plant height, and (C) three environments for ear height. Environments are: Ipiaçú (IP), Nova Mutum (NM), Pato de Minas (PM), Sertanópolis (SE) and Sorriso (SO). Error bars show SD.

**Figure 4:**
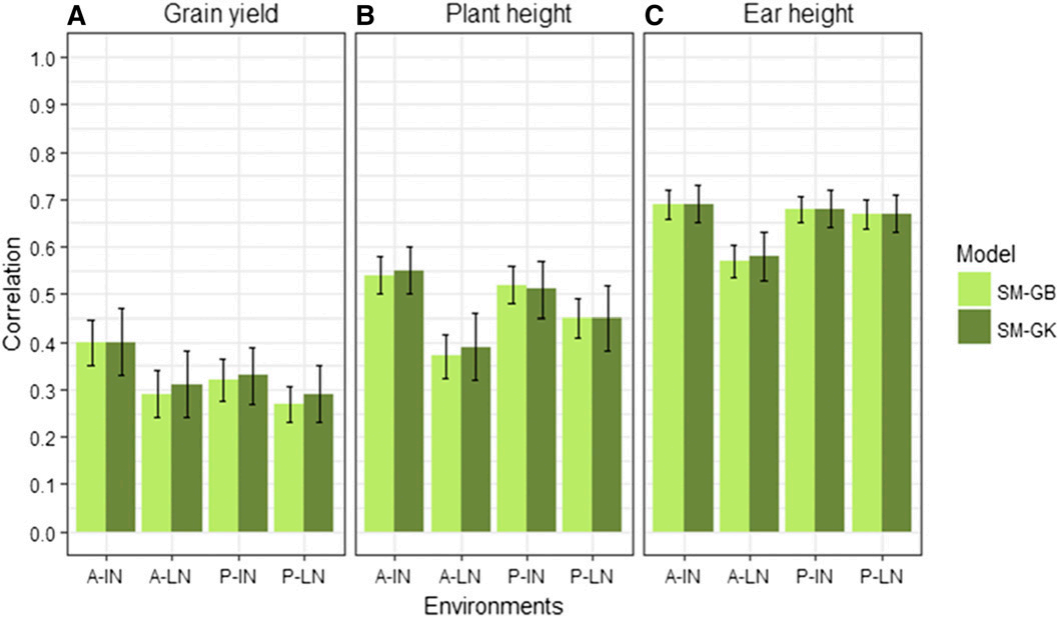
USP data set. Mean correlation between phenotypes and predictions (average of 50 random CV partitions) for single-environment, main genotypic effects model with GBLUP kernel method (SM-GB), and single-environment, main genotypic effects model with GK method (SM-GK) in four environments (horizontal axis) for (A) grain yield, (B) plant height, and (C) ear height. Environments are: Anhumas ideal Nitrogen (A-IN), Anhumas low Nitrogen (A-LN), Piracicaba ideal Nitrogen (P-IN) and Piracicaba low Nitrogen (P-LN). Error bars show SD.

**Figure 5:**
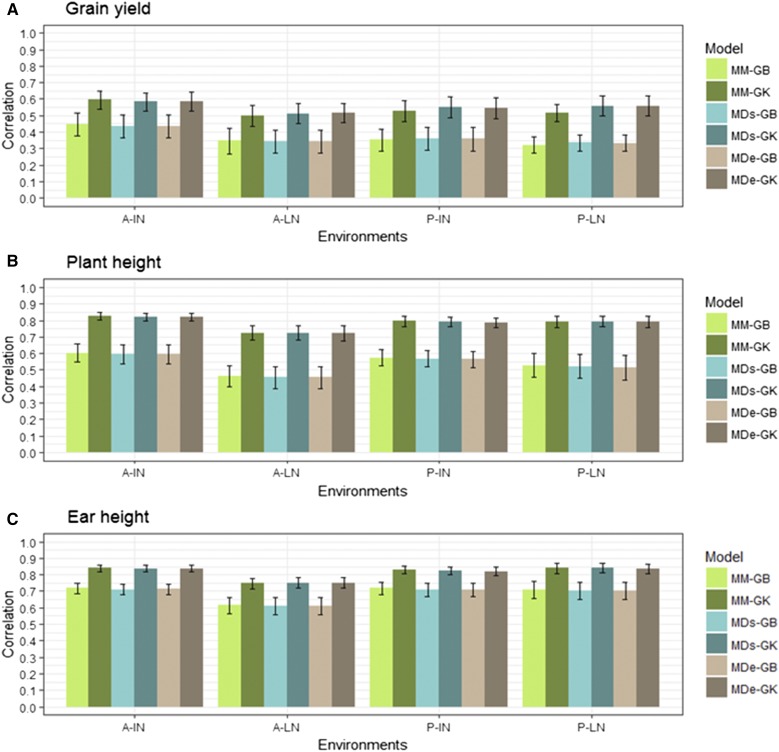
USP data set. Mean correlation between observed and predictive values (average of 50 random CV partitions, CV2) for multi-environment, main genotypic effect model GBLUP kernel (MM-GB), multi-environment, main genotypic effect Gaussian kernel (MM-GK), multi-environment, single variance G×E deviation model GBLUP kernel (MDs-GB), multi-environment, single variance G×E deviation model Gaussian kernel (MDs-GK), multi-environment, environment-specific variance G×E deviation model GBLUP kernel (MDe-GB), multi-environment, environment-specific variance G×E deviation model Gaussian kernel (MDe-GK) in four environments (horizontal axis) for: (A) grain yield, (B) plant height, and (C) ear height. Environments: Anhumas ideal Nitrogen (A-IN), Anhumas low Nitrogen (A-LN), Piracicaba ideal Nitrogen (P-IN), and Piracicaba low Nitrogen (P-LN). Error bars show SD.

Results of the prediction accuracy of the eight model-method combinations for the random CV2 of the two data sets are given in Figure 2, Figure 3, Figure 4, Figure 5, Table 6, and Table 7. Results of the prediction accuracy of the eight model-method combinations for random CV1 of the two data sets are given in Table B1 and Table B2 in Appendix B. Note that, for the single-environment models, prediction accuracy was assessed by 50 random partitions of the data into 80% TRN and 20% TST.

**Table 6:** HEL data set

Model/Environment*^a^*	Single Environment, Main Genotypic Effect Model (SM)		% Change	Multi-Environment, Main Genotypic Effect Model (MM)		% Change	Multi-Environment, Main, Single Variance G×E Deviation Model (MDs)		% Change	Multi-Environment, Environment-Specific Variance G×E Deviation Model (MDe)		% Change
	GB	GK		GB	GK		GB	GK		GB	GK	
Grain yield												
IP	0.64 (0.08)	0.73 (0.06)	14	0.56 (0.09)	0.64 (0.08)	14	0.68 (0.09)	0.81 (0.05)	19	0.66 (0.09)	0.80 (0.05)	21
NM	0.37 (0.09	0.49 (0.09)	32	0.47 (0.08)	0.58 (0.07)	23	0.47 (0.09)	0.63 (0.07)	34	0.43 (0.10)	0.62 (0.06)	44
PM	0.68 (0.05)	0.74 (0.04)	9	0.64 (0.07)	0.70 (0.04)	9	0.69 (0.05)	0.78 (0.03)	13	0.69 (0.05)	0.78 (0.03)	13
SE	0.36 (0.10)	0.46 (0.08)	28	0.25 (0.12)	0.37 (0.11)	48	0.40 (0.10)	0.58 (0.09)	45	0.39 (0.10)	0.58 (0.09)	49
SO	0.40 (0.10)	0.52 (0.07)	30	0.38 (0.08)	0.53 (0.07)	39	0.47 (0.09)	0.69 (0.04)	47	0.47 (0.08)	0.68 (0.04)	45
Plant height												
IP	0.65 (0.08)	0.69 (0.07)	6	0.77 (0.06)	0.80 (0.05)	4	0.78 (0.06)	0.81 (0.05)	4	0.77 (0.06)	0.80 (0.05)	4
PM	0.76 (0.04)	0.79 (0.05)	4	0.79 (0.04)	0.81 (0.04)	3	0.80 (0.04)	0.82 (0.04)	3	0.80 (0.04)	0.82 (0.04)	3
SE	0.74 (0.05)	0.76 (0.05)	3	0.78 (0.04)	0.80 (0.03)	3	0.79 (0.05)	0.82 (0.03)	4	0.79 (0.05)	0.82 (0.03)	4
Ear height												
IP	0.64 (0.08)	0.67 (0.07)	6	0.76 (0.05)	0.76 (0.04)	0	0.76 (0.05)	0.77 (0.04)	1	0.76 (0.05)	0.77 (0.04)	1
PM	0.76 (0.03)	0.78 (0.04)	4	0.78 (0.04)	0.79 (0.03)	1	0.79 (0.03)	0.81 (0.03)	3	0.79 (0.03)	0.81 (0.03)	3
SE	0.69 (0.05)	0.72 (0.05)	3	0.76 (0.05)	0.78 (0.04)	3	0.75 (0.05)	0.78 (0.04)	4	0.75 (0.05)	0.79 (0.04)	5

Mean correlation (for 50 random partitions CV2) for models single-environment, main genotypic effects model with GBLUP kernel method (SM-GB) and single-environment, main genotypic effects model with GK method (SM-GK), multi-environment, main genotypic effect model GBLUP kernel (MM-GB), multi-environment, main genotypic effect GK (MM-GK), multi-environment, single variance G×E deviation model GBLUP kernel (MDs-GB), multi-environment, single variance G×E deviation model GK (MDs-GK), multi-environment, environment-specific variance G×E deviation model GBLUP kernel (MDe-GB), multi-environment, environment-specific variance G×E deviation model GK (MDe-GK) for three traits, grain yield, plant height, and ear height (SD in parentheses).

a Environments: IP, Ipiaçú; NM, Nova Mutum; PM, Pato de Minas; SE, Sertanópolis; SO, Sorriso.

**Table 7:** USP data set

Model/Environment*^a^*	Single Environment, Main Genotypic Effect Model (SM)		% Change	Multi-Environment, Main Genotypic Effect Model (MM)		% Change	Multi-Environment, Main, Single Variance G×E Deviation Model (MDs)		% Change	Multi-Environment, Environment-Specific Variance G×E Deviation Model (MDe)		% Change
	GB	GK		GB	GK		GB	GK		GB	GK	
Grain yield												
P-LN	0.27 (0.04)	0.29 (0.06)	7	0.32 (0.05)	0.51 (0.05)	59	0.34 (0.05)	0.56 (0.06)	65	0.33 (0.05)	0.56 (0.06)	70
P-IN	0.32 (0.04)	0.33 (0.06)	3	0.35 (0.07)	0.53 (0.07)	51	0.36 (0.07)	0.55 (0.06)	53	0.36 (0.07)	0.55 (0.06)	53
A-LN	0.29 (0.05)	0.31 (0.07)	7	0.35 (0.08)	0.50 (0.06)	43	0.34 (0.07)	0.51 (0.06)	50	0.34 (0.07)	0.51 (0.06)	50
A-IN	0.40 (0.05)	0.40 (0.07)	0	0.44 (0.07)	0.59 (0.05)	34	0.43 (0.07)	0.58 (0.06)	35	0.43 (0.07)	0.59 (0.06)	37
Plant height												
P-LN	0.45 (0.04)	0.45 (0.07)	0	0.53 (0.07)	0.79 (0.03)	49	0.52 (0.07)	0.79 (0.03)	52	0.51 (0.07)	0.79 (0.03)	55
P-IN	0.52 (0.04)	0.51 (0.06)	−2	0.57 (0.05)	0.79 (0.03)	39	0.57 (0.05)	0.79 (0.03)	39	0.56 (0.05)	0.79 (0.03)	41
A-LN	0.37 (0.05)	0.39 (0.07)	5	0.46 (0.07)	0.72 (0.04)	57	0.46 (0.07)	0.72 (0.04)	57	0.45 (0.07)	0.72 (0.05)	61
A-IN	0.54 (0.04)	0.55 (0.05)	2	0.60 (0.05)	0.83 (0.02)	38	0.60 (0.06)	0.82 (0.02)	37	0.60 (0.06)	0.82 (0.02)	37
Ear height												
P-LN	0.67 (0.03)	0.67 (0.04)	0	0.71 (0.05)	0.84 (0.03)	18	0.70 (0.05)	0.84 (0.03)	20	0.70 (0.05)	0.84 (0.03)	20
P-IN	0.68 (0.03)	0.68 (0.04)	0	0.72 (0.04)	0.83 (0.02)	15	0.71 (0.04)	0.82 (0.02)	15	0.71 (0.04)	0.82 (0.03)	15
A-LN	0.57 (0.03)	0.58 (0.05)	2	0.61 (0.05)	0.75 (0.03)	23	0.61 (0.05)	0.75 (0.03)	23	0.61 (0.05)	0.75 (0.03)	23
A-IN	0.69 (0.03)	0.69 (0.04)	0	0.72 (0.03)	0.84 (0.02)	17	0.71 (0.03)	0.84 (0.02)	18	0.71 (0.03)	0.84 (0.02)	18

Mean correlation (for 50 random partitions CV2) for models single-environment, main genotypic effects model with GBLUP kernel method (SM-GB) and single-environment, main genotypic effects model with GK method (SM-GK), multi-environment, main genotypic effect model GBLUP kernel (MM-GB), multi-environment, main genotypic effect GK (MM-GK), multi-environment, single variance G×E deviation model GBLUP kernel (MDs-GB), multi-environment, single variance G×E deviation model GK (MDs-GK), multi-environment, environment-specific variance G×E deviation model GBLUP kernel (MDe-GB), multi-environment, environment-specific variance G×E deviation model GK (MDe-GK) for three traits, grain yield, plant height, and ear height (SD in parentheses)

a Environments: A-IN, Anhumas ideal N, A-LN, Anhumas low N, P-IN, Piracicaba ideal N; and P-LN, Piracicaba low N

### HEL data set

#### Single-environment:

Results for CV2 for GY showed that the prediction accuracy of the SM-GK model-method was higher than for SM-GB in all environments (Figure 2). For GY in environment IP, prediction accuracies were 0.64 for SM-GB and 0.73 for SM-GK, while in environment PM, prediction accuracies were 0.68 for SM-GB and 0.74 for SM-GK (Table 6). The percent change in accuracy for single-environment GB *vs.* single-environment GK for GY ranged from 9 to 32% for the PM and NM environments, respectively. For trait PH, prediction accuracies were high: >0.65 for SM-GB and >0.69 for SM-GK. For trait EH, prediction accuracies were >0.64 for SM-GB and >0.67 for SM-GK; the increase in prediction accuracy ranged from 3 to 6% for traits EH and PH (Table 6).

#### Multi-environment:

For GY, the best model-methods for CV2 were MDs-GK and MDe-GK for the five environments, followed by MDs-GB in the IP environment, MM-GK in the NM, PM, and SO environments and by MDs-GB and MDe-GB in the SE environment (Figure 3). For trait GY, the percent change in accuracy for MM-GB *vs.* MM-GK was high, ranging from 9% in PM to 48% in SE environment; for MDs-GB *vs.* MDs-GK, the % change ranged from 13% in PM to 47% in the SO environment; and for MDe-GB *vs.* MDe-GK, the % change ranged from 13% in PM to 49% in SE environment (Table 6). These results show that, for trait GY, the increase in prediction accuracy was high using the GK method. For less complex traits PH and EH, the percent change of GK over GB for all models showed a small increase in prediction accuracy, with the percent change ranging from 1 to 6%; there was no difference for trait EH in the IP environment.

For GY, models MDs-GK and MDe-GK were, for CV2, the models that had the best prediction accuracy in all environments, with values ranging from 0.58 in SE to 0.81 in the IP environment. These values were higher for a complex trait like GY (Figure 3). For the PH trait, MDs-GK and MDe-GK were the best models, with a prediction accuracy of 0.81–0.82 for all environments; for trait EH, these values ranged from 0.77 in IP to 0.81 in PM. In general, MDs models had similar results across environments.

Prediction accuracy for random CV1 decreased (Table B1, Appendix B) as compared with those computed for CV2 for all traits and models. For example, for environment IP, MM-GB in CV2 gave a correlation of 0.56, whereas for CV1, prediction accuracy was 0.48, that is, an 8% decrease in accuracy, whereas, for environment NM, the decrease is ∼10%. Similar decreases in accuracy from CV1 to CV2 were found for other traits and model-method combinations. However, for GY, models with GK showed a greater increase in prediction accuracy over models with GB under CV1 (Table B1, Appendix B) than the increases achieved by those models under CV2 (Table 6). The % change in prediction accuracy of CV1 *vs.* CV2 for models with GK *vs.* GB did not change for traits PH and EH.

#### Summary of results:

Prediction accuracy of trait GY in the five testing environments increased from 9 to 48% for models with method GK compared to models with method GB. For GY, increases in prediction accuracy of environments from multi-environment models MM, MDs, and MDe with GK *vs.* MM, MDs, and MDe with GB tended to be higher than those achieved by the single-environment model (SM-GK *vs.* SM-GB). For traits PH and EH in three environments, the increase in prediction accuracy of method GK over method GB ranged only from 0 to 6%, and increases in prediction accuracy from multi-environment models MM, MDs, and MDe with GK *vs.* MM, MDs, and MDe with GB tended to be lower than those achieved by the single-environment model (SM-GK *vs.* SM-GB).

Although prediction accuracy for all model-method combinations decreased in CV1 *vs.* CV2, the % change in the prediction accuracy of models with GK *vs.* models GB did not change and even increased for GY.

### USP data set

#### Single-environment:

Table 7 shows the correlations between observed and predicted values for all models in the USP data set. In general, for traits GY, PH, and EH, all prediction accuracies of the single-environment models were similar using the GB or GK methods (Figure 4). In these cases, the percentage change in accuracy for SM-GB *vs.* SM-GK was 7% (GY) in environment P-LN and 5% (PH) in environment A-LN. For GY, the prediction accuracy in the A-IN environment (0.40 for SM-GB and SM-GK) was greater than in other environments. Gains in prediction accuracy of SM-GK over SM-GB for trait GY in this data set are much lower than those achieved in the HEL data set.

#### Multi-environment:

Between the GB and GK methods, there were large increases in prediction accuracy using the GK method, especially for traits GY and PH (Figure 5). For trait GY, the MM model gave a high percentage change in accuracy for GB *vs.* GK ranging from 34 to 59%. In the MDs model, the percentage change ranged from 35 to 65%, and for the MDe model, the percentage change ranged from 37 to 70% (Table 7).

For PH, the increase using the GK method was similar to those found for the GY trait. The percentage change for the MM model with GB *vs.* GK ranged from 38% (A-IN and P-IN) to 57% (A-LN). For the MDs models, the percentage change ranged from 37% (A-IN) to 61% (A-LN), and, for the MDe models, the percentage change ranged from 37% (A-IN) to 51% (A-LN). For trait EH, the increase was smaller, ranging from 15 to 23% for the GBLUP *vs.* the GK method. The MM and MDs models showed similar correlations when compared using the same kernel method. Models MDs-GK and MDe-GK had a clear and sustainable increase in prediction accuracy over their counterparts using the GB kernel, MDs-GB, and MDe-GB.

The prediction accuracy of random CV1 decreased (Table B2, Appendix B) compared to the accuracy obtained with CV2 for all traits and models. In general, compared with other models, models MDs and MDe decreased the accuracy of CV1 *vs.* CV2 more; this was more pronounced for GY in some environments than in other environments. Also, the decrease in the accuracy of CV1 *vs.* CV2 was higher for GY than for PH and EH.

#### Summary of results:

The prediction accuracy of trait GY in four environments increased from 34 to 70% for multi-environment models MM, MDs, and MDe with GK *vs.* MM, MDs, and MDe with GB. These gains in accuracy were much higher than those achieved by single-environment SM-GK over SM-GB (from 0 to 7%). Similar patterns were found for traits PH and EH, where the increase in prediction accuracy of method GK over method GB ranged from −2 to 5% for the single-environment model, SM, and drastically increased for multi-environment models MM, MDs, and MDe with GK *vs.* MM, MDs, and MDe with GB, ranging from 15 to 57%.

Prediction accuracy for all model-method combinations decreased in CV1 *vs.* CV2 and the % change in accuracy of models with GK *vs.* models with GB decreased for all three traits. The increase in accuracy of models with GK *vs.* models with GB did not occur under the CV1 design.

## Discussion

Several studies have documented the benefits of using a nonlinear Gaussian kernel in multi-environment models for capturing small complex interactions among markers, and increasing prediction accuracy (Gianola *et al.* 2006, 2014; Crossa *et al.* 2010; Pérez-Rodríguez *et al.* 2013; Pérez-Elizalde *et al.* 2015; Cuevas *et al.* 2016a,b). In this research, genome-enabled prediction accuracy was studied in two different hybrid maize data sets using multi-environment models with the GBLUP and GK methods. In this context, we proposed including the GK in the model of Jarquín *et al.* (2014), which accounts for environment information without and with interaction terms. Prediction accuracy was obtained using traits with different genetic architecture and heritability values that ranged from low to high.

### Prediction accuracy differences in datasets, methods, CV designs and G×E

We compared the single-environment model with two G×E models. The first G×E model can accommodate environmental covariables (Jarquín *et al.* 2014) that were not available in this study; the second G×E model (López-Cruz *et al.* 2015) accommodates environment main effects and environment-specific variances; both models used the GB and GK methods proposed by Cuevas *et al.* (2016a). These two G×E models have demonstrated good prediction accuracy when used in genomic-enabled prediction studies (López-Cruz *et al.* 2015; Zhang *et al.* 2015; Crossa *et al.* 2016a; Saint Pierre *et al.* 2016). We found that GK methods improved the prediction ability of all single-environment and multi-environment models for CV2 design for all traits in HEL and USP datasets. Prediction of all traits with multi-environment models incorporating the G×E term, and the GK method gave better prediction accuracy (especially for complex traits such as GY) than the other model-methods. The increases in prediction accuracy using models with GK under random (CV2) are also reflected under random cross-validation 1 (CV1) for HEL data set, but, to a lesser extent due to the difficulty of borrowing information of unobserved (unphenotyped) lines in all environments. Similar decreases in prediction accuracy were found by López-Cruz *et al.* (2015), when attempting to predict wheat lines in untested (unobserved) environments under the CV1 random partition scheme.

Furthermore, for trait GY, differences in CV1 *vs.* CV2 for GB and GK methods for the two data sets might also be due to other factors such as:

1. Differences in dataset repeatability, higher in HEL data sets (0.81, 0.60, 0.86, 0.69, and 0.78 for each of the five environments) than in USP (0.22, 0.42, 0.19, and 0.42 for each of the four environments) (Table 2 and Table 3, respectively) due to better quality of the replicated trials in HEL than the unreplicated trials in USP;
2. More opportunity to borrow information from the close relatives hybrids in HEL (genetic diversity = 0.175) than from the less related hybrids included in USP dataset (genetic diversity = 0.372). For *h* = 1 (bandwidth parameter) the GK is a direct function of the squared Euclidean distance between hybrids based on markers ($d_{ii^{\prime }}$). Thus, for a set of unrelated hybrids $d_{ii^{\prime }}\to$ large and K→**I** (identity matrix), *i.e.*, the GK weight more heavily the within (own) hybrid information compared to the between hybrid (from relatives) information than GB (the marker information is of no use) (de los Campos *et al.* 2010). On the other hand, for a set of related hybrids then $d_{ii^{\prime }}\to$ 0 and K→**1** (matrix of ones), *i.e.*, marker information given by the GK weight equally or less the own hybrid information compared to information from relatives given by GB. Therefore, we speculate that, for related hybrids of HEL dataset, GK gives heavier weights to the within hybrid as well as between hybrids than the GB method does, that is why for GY, CV1 and CV2 for the multi-environment models gave very good increase in prediction accuracy over the GB;
3. For CV2, G×E modulates both values (within and between hybrid information); hybrids in USP (doubled the genetic diversity) less related than those from HEL data set, then it is expected negligible values between hybrids, and similar values assign by GK and GB to within hybrid information; for CV2, predictions accuracy depends, to a great extent, on the correlations between environments.

In summary, for CV2, for the prediction accuracy of GY in the HEL dataset, the method GK weights own, and between hybrid, performance as well as the relationship between environment (G×E modeled by MDs y MDe), whereas, for USP, GK weights only the own hybrid performance and the relationship between environment becomes more important that in the HEL data set.

In the studies of López-Cruz *et al.* (2015) and Cuevas *et al.* (2016a), the data were balanced in the sense that all the individuals were included at the same time in all the environments. On the contrary, in this study, we had a great deal of imbalance because different numbers of maize hybrids were included in different environments; this was more pronounced in the HEL data set than in the USP data set, and different R scripts were necessary for implementing the model–method combinations.

### Prediction accuracy using linear and nonlinear kernel methods

According to Gianola *et al.* (2014), GK has better predictive ability and a more flexible structure than GBLUP. Another point is that GK can capture nonadditive effects between markers. Jiang and Reif (2015) evaluated maize data sets and investigated whether the prediction accuracy across connected biparental families can be increased by modeling additive × additive epistasis; the authors found that the prediction accuracy of RKHS (including epistasis) was superior to that of GBLUP (ignoring epistasis). Our study clearly shows a large increase in predictive ability when the G×E model and the GK method are combined for all traits, but mainly for GY (both data sets) and PH (USP data set). This indicates that, to increase predictive ability, it is important to consider the nonadditive effect (*i.e.*, epistasis) as the genetic relatedness across connected populations.

There are different choices for computing kernel functions: for example, linear kernel matrices incorporate only additive effects of the markers, polynomials kernels of different orders might incorporate different degrees of marker interactions, and the Gaussian kernel function uses complex epistatic marker interaction (Akdemir and Jannink 2015). In GS, additive kernels of the GBLUP type are employed when predicting breeding values, whereas, when attempting to predict genetic values, GKs would be more appropriate. Akdemir and Jannink (2015) demonstrated that epistatic marker effects in local regions of the chromosome with low recombination are stable through generations, and offer the opportunity to exploit epistasis for improving genomic-enabled prediction accuracy; the authors defined local kernels in regions of the genome and calculated separate kernels for each region. The results shown in this study clearly showed the benefit of exploiting these local epistatic effects captured in the GK, and their interaction with environments.

The flexible structure of the MDs and MDe models is important, especially when combined with kernels that capture nonadditive effects, as previously proven. In general, in the two maize data sets, the MDs-GK and MDe-GK models had better prediction accuracy than other models. The increase was not higher due to the occurrence of intermediate-to-high positive correlations between the analyzed environments, resulting in low G×E. These results corroborate those obtained by López-Cruz *et al.* (2015) and Cuevas *et al.* (2016a), who observed that the intensity of the environmental correlation is related to the proportion of the genomic variance explained by the genetic main effects of markers across environments and genetic-specific effects of markers in environments.

### Better fit of the G×E GK models

For GY of the HEL data set, the better fit of the model-method MDe-GB over the MDs-GB and MM-GB models is evident in their residual variance components: 0.227, 0.230, and 0.336, respectively. Similarly, for GY, the better fit of model MDe-GK over the MDs-GK and MM-GK models is also evident in their residual variance components: 0.093, 0.107, and 0.273, respectively. These trends in the residuals of the models are also found in traits PH and EH. The residuals of GK models were lower than those of the GBLUP models, indicating the better fit of the nonlinear *vs.* the linear kernel methods.

For GY of the USP data set, similar trends in the residuals of model-method MDe-GB *vs.* the MDs-GB and MM-GB models were found: 0.840, 0.836, and 0.864, respectively. Similar clear trends in the residuals of these models were found for traits PH and EH. These patterns are also clear for the residuals of GK model-methods MDe-GK, MDs-GK, and MM-GK for trait EH, but not for trait PH.

### Prediction accuracy using multi-environment models

In this study, all pairwise correlations between environments were high and positive. This is important, because the G×E model of López-Cruz *et al.* (2015) has the limitation of better and more efficient prediction when applied to subsets of environments that have positive and similar correlations (Crossa *et al.* 2016b; Cuevas *et al.* 2016a). In positively correlated environments, the main marker effects are the most influential components when predicting genetic values; their variance component is high, and this produces better prediction accuracy than the single-environment model (López-Cruz *et al.* 2015; Cuevas *et al.* 2016a). Environments with intermediate or high positive correlations indicate little G×E interaction. Thus, the model reacts by reducing the specific marker effect. A correlation that is negative or close to zero implies strong G×E interaction, making it difficult to predict one environment based on information from another. With this, the model reduces the main effect of the marker, and increases the specific effects. To work around the limitations of the G×E models, Cuevas *et al.* (2016b) developed multi-environment Bayesian genomic models that allow an arbitrary genetic covariance structure between environments, because an unstructured covariance matrix was used, and its parameters were estimated from the data.

In this study, the prediction of multi-environment models was assessed by applying the CV strategy called CV2 in Burgueño *et al.* (2012) and (Cuevas *et al.* 2016a), where some lines are represented in some environments but not in others. This CV2 validation strategy performed better than CV strategy CV1 (where lines have not been evaluated in any field trials) proposed by Burgueño *et al.* (2012), when applied in multi-environment models (Jarquín *et al.* 2014; López-Cruz *et al.* 2015; Crossa *et al.* 2016b; Saint Pierre *et al.* 2016).

When introducing interaction effects, models MDs and the MDe showed increases in prediction accuracy for GY in most environments for the HEL and USP data sets over the single-environment model. Furthermore, models with GK had superior prediction accuracy than GB models. For traits PH and EH in both data sets, not much increase in prediction accuracy of MDs and MDe models was achieved over the single-environment model. These results were expected because the genetic architecture of traits PH and EH is less complex than that of GY and less influenced by environmental factors. When main marker effects and interaction effects are introduced in the model using covariance structures, this improves prediction accuracy.

The increase in accuracy when the interaction term for complex traits was included concurs with the findings of Crossa *et al.* (2016a), where increases in prediction accuracy were achieved by including dense molecular markers and G×E in a set of Mexican and Iranian landraces. Zhang *et al.* (2015) also used multi-environment models incorporating G×E, and obtained an increase in prediction accuracy of several maize biparental populations. Similarly, Saint Pierre *et al.* (2016) evaluated spring wheat lines and introduced other environmental covariates in the Jarquín’s model, showing that the prediction models gave better predictions using random CV. Recently, results of extensive analyses of spring wheat trials across international environments conducted for several years in South and West Asia, North Africa, and Mexico showed a consistent increase in the genomic prediction accuracy of the MDs-GB model over the MM-GB and SM-GB models (Sukumaran *et al.* 2017). One limitation of the data used in this study is that only 1 yr was available for assessing the genomic-enabled accuracy of the various models.

Our results show that predictions with medium-to-high accuracy for GS programs can be expected in environments with low-to-high heritability. Despite the low heritability of GY in P-LN and A-LN environments, the results were similar to results in other environments that have medium-to-high heritability. But, in general, for the USP data set, prediction accuracy of all target traits under stress conditions was lower than under ideal nitrogen conditions, while in stress environments, heritability was lower than under ideal nitrogen conditions. These results agree with those obtained by Zhang *et al.* (2015), who found lower genomic prediction accuracy under water stress conditions than under ideal conditions, especially for complex traits such as GY.

### Conclusions

Incorporating the GK method into the model of Jarquín *et al.* (2014) increased prediction accuracy as compared to the model used with the linear kernel GBLUP; these results were found in both data sets with the models used in this study: MM across environments, MDs, and MDe. Other results of the application of eight model-method combinations between four models (SM, MM, MDs, and MDe) and two kernel methods (GBLUP and Gaussian kernel) in two extensive maize data sets show that (1) genomic models incorporating G×E interaction had higher prediction accuracy than single-environment models; and (2) models with nonlinear Gaussian kernel had higher prediction accuracy than models with linear kernel GBLUP. The model-method combinations with the highest prediction accuracy were MDe-GK and MDs-GK. Results of this study indicated that by employing appropriate statistical genomic-enabled prediction models the researchers and plant breeders can improve the prediction of hybrids that were not evaluated in several environments. Random CV2 mimics sparse (incomplete) testing that allow to safe resources to the breeding program while improving prediction accuracy. Further research is required to compare the genomic-enabled G×E kernel prediction models used in this study with the models recently developed by Cuevas *et al.* (2016b), who studied genomic-enabled G×E models by means of Kronecker products applied to unstructured covariance matrices. It is also necessary to develop efficient computing software for fitting the structures considered in the models of this study while maintaining the inferential advantages of the Markov Chain Monte Carlo.

## Appendix A

In Box A1, we provide simplified scripts that can be used to obtain the GBLUP and GK matrix, to be used in the multi-environment models used in this study. The example uses the following R-object:

- X (n×p) a genomic marker matrix. The line IDs can be retrieved using either rownames(X) or SNP IDs in colnames(X).

In the examples used to fit MM, MDs and MDe models, an R-object is necessary as matrix (pheno_geno) compound by three vectors of Location_id, Germplasm_id and Y, where:

pheno_geno$Location_id vector is compound by environment information
pheno_geno$Germplasm_id vector is compound by germplasm names
pheno_geno$Y is a numeric and standardized vector with yield records in all environments

In Box A2 we give simplified scripts that can be used to fit models MM, MDs or MDe

## Appendix B

Box A1. An Example of How to Obtain GBLUP (GB) and GK Matrix X<-scale(X,center=TRUE,scale=TRUE) # G-BLUP kernel (GB) GB<-tcrossprod(X)/ncol(X) # Gaussian Kernel (GK) dist<-as.matrix(dist(X))^2 GK<-exp(-dist/median(dist))

Box A2. An Example of How to fit MDs model with cross-validation scheme CV2 # Loading objects needed for MM, MDs and MDe models library(BGLR) env<-c(1,2,3,4) ### Choose environment K<-GK #### Choose kernel GK or GB model<- “MDs” ### Choose model “MM”or”MDs”or”MDe” CV<- 2 ### Choose CV=1 (CV1), CV=2 (CV2) nEnv<-length(env) pheno_geno1<-pheno_geno[pheno_geno$Location==env[1],] i<-2 while (i <=nEnv){ pheno_geno1<-rbind(pheno_geno1,pheno_geno[pheno_geno$Location==env[i],]) i<-i+1 } envID<-as.factor(pheno_geno1[,1]) IDs<-as.character(unique(pheno_geno1$Germplasm_id)) IDy<-pheno_geno1$Germplasm_id Y <- as.numeric(pheno_geno1[,3]) Y <- scale(Y,center=TRUE,scale=TRUE) names(Y)<-IDy nEnv <- length(env) K<-K[IDs,IDs] phenol_geno1$ Germplasm_id<-factor(x= phenol_geno1$ Germplasm_id,levels=rownames(K),ordered=TRUE) Zg <- model.matrix(∼factor(pheno_geno1$Germplasm_id)-1) # genotype design matrix Ze <- model.matrix(∼factor(pheno_geno1$Location)-1) # environment design matrix K1 <- Zg%*%K%*%t(Zg) # CV scheme fit set.seed(12345) nFolds <- 5 partitions <- 10 if (CV == 1) { mfold <- matrix(NA, ncol = partitions, nrow = length(IDs)) sets <- matrix(NA, nrow = nrow(pheno_geno1), ncol = partitions) for (s in 1:partitions) { mfold[, s] <- sample(1:nFolds, size = length(IDs), replace = TRUE) for (i in 1:nrow(pheno_geno1)) { sets[i, s] <- mfold[which(IDs == pheno_geno1$Germplasm_id[i]), s] } } } if (CV == 2) { sets <- matrix(NA, nrow = nrow(pheno_geno1), ncol = partitions) for (s in 1:partitions) { for (i in IDs) { tmp = which(IDy == i) ni = length(tmp) tmpFold <- sample(1:nFolds, size = ni, replace = ni > nFolds) sets[tmp, s] <- tmpFold } } } trn_tst<-matrix(nrow=nrow(pheno_geno1),ncol=nFolds,NA) NA.y <- matrix(Y, ncol = nFolds, nrow = length(Y)) # Fitting MM model if (model==”MM”) { ETA<-list(ENV=list(X=Ze,model=”FIXED”), Grm=list(K=K1,model=”RKHS”))} # Fitting MDs model if(model==”MDs”){ ZEZE<-tcrossprod(Ze) K2<-K1*ZEZE ETA<-list(ENV=list(X=Ze,model=”FIXED”), Grm=list(K=K1,model=”RKHS”), EGrm=list(K=K2,model=”RKHS”))} # Fitting MDe model if (model==”MDe”){ ETA<-list(ENV=list(X=Ze,model=”FIXED”), Grm=list(K=K1,model=”RKHS”)) for(k in 1:nEnv){ ZEE<-matrix(0,nrow=nrow(Ze),ncol=ncol(Ze)) ZEE[,k]<-Ze[,k] ZEEZ<-(ZEE%*%t(Ze)) K3<-K1*ZEEZ ETA[[k+2]]<-list(K=K3,model=’RKHS’) }} COR<-matrix(0,nrow=nEnv,ncol=(nFolds*partitions)) for (s in 1:partitions) { NA.y <- matrix(Y, ncol = (nFolds), nrow = length(Y)) for(i in 1:ncol(trn_tst)){ trn_tst[,i]<-ifelse(sets[,s]==i,’tst’,’trn’) NA.y[,i][(trn_tst[,i]==”tst”)]<-NA A <- as.matrix(NA.y[,i]) fm <- BGLR(y=A,ETA=ETA,nIter=10000,burnIn=1000,thin=2) for(g in 1:length(env)){ tst1 <- which(is.na(fm$y[envID==env[g]])) COR[g,((s-1)*nFolds+i)] <- cor(Y[envID==env[g]][tst1],fm$yHat[envID==env[g]][tst1]) } } }

**Table B1:** HEL data set

Model/Environment*^a^*	Multi-Environment, Main Genotypic Effect Model (MM)		% Change	Multi-environment, Main, Single Variance G×E Deviation Model (MDs)		% Change	Multi-Environment, Environment-Specific Variance G×E Deviation Model (MDe)		% Change
	GB	GK		GB	GK		GB	GK	
Grain yield									
IP	0.48 (0.12)	0.60 (0.08)	25	0.65 (0.09)	0.75 (0.05)	15	0.64 (0.09)	0.75 (0.05)	17
NM	0.36 (0.10)	0.48 (0.09)	33	0.41 (0.08)	0.55 (0.07)	34	0.41 (0.08)	0.54 (0.07)	32
PM	0.60 (0.06)	0.70 (0.05)	17	0.68 (0.04)	0.75 (0.04)	10	0.68 (0.04)	0.75 (0.04)	10
SE	0.17 (0.13)	0.28 (0.11)	65	0.32 (0.11)	0.52 (0.08)	63	0.31 (0.11)	0.51 (0.09)	65
SO	0.27 (0.11)	0.42 (0.10)	56	0.39 (0.08)	0.56 (0.07)	44	0.38 (0.08)	0.56 (0.07)	47
Plant height									
IP	0.72 (0.07)	0.74 (0.06)	3	0.73 (0.06)	0.75 (0.06)	3	0.73 (0.06)	0.75 (0.06)	3
PM	0.75 (0.06)	0.78 (0.05)	4	0.76 (0.06)	0.79 (0.05)	4	0.76 (0.06)	0.79 (0.05)	4
SE	0.74 (0.06)	0.76 (0.05)	3	0.75 (0.06)	0.77 (0.05)	3	0.75 (0.06)	0.77 (0.05)	3
Ear height									
IP	0.71 (0.06)	0.72 (0.06)	1	0.72 (0.06)	0.73 (0.06)	1	0.72 (0.06)	0.72 (0.06)	0
PM	0.74 (0.06)	0.76 (0.06)	3	0.76 (0.06)	0.78 (0.05)	3	0.76 (0.06)	0.78 (0.05)	3
SE	0.72 (0.06)	0.73 (0.05)	1	0.72 (0.06)	0.73 (0.05)	1	0.72 (0.06)	0.73 (0.05)	1

Mean correlation (for 50 random partitions, CV1) for models multi-environment, main genotypic effect model GBLUP kernel (MM-GB), multi-environment, main genotypic effect GK (MM-GK), multi-environment, single variance G×E deviation model GBLUP kernel (MDs-GB), multi-environment, single variance G×E deviation model GK (MDs-GK), multi-environment, environment-specific variance G×E deviation model GBLUP kernel (MDe-GB), multi-environment, environment-specific variance G×E deviation model GK (MDe-GK) for three traits, grain yield, plant height, and ear height (SD in parentheses).

a Environments: IP, Ipiaçú; NM, Nova Mutum; PM, Pato de Minas; SE, Sertanópolis; and SO, Sorriso.

**Table B2:** USP data set

Model/Environment*^a^*	Multi-Environment, Main Genotypic Effect Model (MM)		% Change	Multi-Environment, Main, Single Variance G×E Deviation Model (MDs)		% Change	Multi-Environment, Environment-Specific Variance G×E Deviation Model (MDe)		% Change
	GB	GK		GB	GK		GB	GK	
Grain yield									
P-LN	0.26 (0.06)	0.27 (0.06)	4	0.29 (0.07)	0.29 (0.07)	0	0.29 (0.07)	0.28 (0.07)	−3
P-IN	0.31 (0.07)	0.30 (0.06)	−3	0.34 (0.07)	0.33 (0.06)	−3	0.34 (0.07)	0.33 (0.06)	−3
A-LN	0.30 (0.08)	0.28 (0.07)	−7	0.31 (0.07)	0.27 (0.07)	−13	0.31 (0.07)	0.28 (0.07)	−10
A-IN	0.40 (0.06)	0.39 (0.06)	−3	0.42 (0.05)	0.41 (0.05)	−2	0.42 (0.05)	0.41 (0.05)	−2
Plant height									
P-LN	0.47 (0.06)	0.45 (0.06)	−4	0.47 (0.06)	0.44 (0.06)	−6	0.47 (0.06)	0.44 (0.06)	−6
P-IN	0.52 (0.06)	0.48 (0.06)	−8	0.52 (0.06)	0.49 (0.06)	−6	0.52 (0.06)	0.49 (0.06)	−6
A-LN	0.40 (0.07)	0.41 (0.07)	3	0.40 (0.07)	0.40 (0.06)	0	0.40 (0.07)	0.40 (0.06)	0
A-IN	0.55 (0.05)	0.51 (0.05)	−7	0.55 (0.05)	0.52 (0.05)	−5	0.55 (0.05)	0.52 (0.05)	−5
Ear height									
P-LN	0.68 (0.04)	0.67 (0.04)	−1	0.68 (0.04)	0.67 (0.04)	−1	0.68 (0.04)	0.66 (0.04)	−3
P-IN	0.69 (0.04)	0.68 (0.04)	−1	0.69 (0.04)	0.68 (0.04)	−1	0.69 (0.04)	0.68 (0.04)	−1
A-LN	0.59 (0.06)	0.59 (0.05)	0	0.59 (0.06)	0.58 (0.05)	−2	0.59 (0.06)	0.58 (0.05)	−2
A-IN	0.69 (0.03)	0.68 (0.03)	−1	0.69 (0.03)	0.68 (0.03)	−1	0.69 (0.03)	0.68 (0.03)	−1

Mean correlation (for 50 random partitions, CV1) for models multi-environment, main genotypic effect model GBLUP kernel (MM-GB), multi-environment, main genotypic effect GK (MM-GK), multi-environment, single variance G×E deviation model GBLUP kernel (MDs-GB), multi-environment, single variance G×E deviation model GK (MDs-GK), multi-environment, environment-specific variance G×E deviation model GBLUP kernel (MDe-GB), multi-environment, environment-specific variance G×E deviation model GK (MDe-GK) for three traits, grain yield, plant height, and ear height (SD in parentheses).

a Environments: Anhumas ideal N (A-IN), Anhumas low N (A-LN), Piracicaba ideal N (P-IN) and Piracicaba low N (P-LN).
